# Optimal Principle of Bone Structure

**DOI:** 10.1371/journal.pone.0028868

**Published:** 2011-12-16

**Authors:** Yifang Fan, Yubo Fan, Zhiyu Li, Mushtaq Loan, Changsheng Lv, Zhang Bo

**Affiliations:** 1 Center for Scientific Research, Guangzhou Institute of Physical Education, Guangzhou, People's Republic of China; 2 Key Laboratory for Biomechanics and Mechanobiology of Ministry of Education, School of Biological Science and Medical Engineering, Beihang University, Beijing, People's Republic of China; 3 College of Foreign Studies, Jinan University, Guangzhou, People's Republic of China; 4 International School, Jinan University, Guangzhou, People's Republic of China; Ohio State University, United States of America

## Abstract

Bone modeling and remodeling is an optimization process where no agreement has been reached regarding a unified theory or model. We measured 384 pieces of bone *in vivo* by 64-slice CT and discovered that the bone's center of mass approximately superposes its centroid of shape. This phenomenon indicates that the optimization process of non-homogeneous materials such as bone follows the same law of superposition of center of mass and centroid of shape as that of homogeneous materials. Based upon this principle, an index revealing the relationship between the center of mass and centroid of shape of the compact bone is proposed. Another index revealing the relationship between tissue density and distribution radius is followed. Applying these indexes to evaluate the strength of bone, we have some new findings.

## Introduction

The optimization of bone's size, shape and structure is a physical process [1], [2], [3], [4] and the process is an adaptive response [3], [5], [6]. The adaptive responses of bone tissue generated by activities such as bone modeling and remodeling maximize its bearing load [7]. However, it remains uncertain what principles of mechanics these adaptive changes of bone follow.

Wolff's law [8] on bone's adaptive changes served as a prelude to the study of bone modeling and remodeling. Wolff's law was refined by Frost who promoted his Mechanostat theory [9], describing the bone's transformation on the tissue level. An ideal description of its mechanism should be studied from the perspectives of cell, molecule or gene [10] though no matter from which perspective, no agreement on a unified theory or model has been reached [11], [12]. What's more, the complexity of bone's loading has brought difficulties (such as the target function or constraint equation involved in the target optimization analysis) in defining when the minimal material can sustain the maximal loading [3], [13], [14].

We assume that the optimization process of the non-homogeneous bone follows the same law of superposition of its center of mass (COM) and centroid of shape (COS) of the homogeneous material. A spiral CT scanning with an accuracy of sub-millimeter is conducted to 32 feet *in vivo*. An analysis to the positional relationship between the COM and COS of 384 pieces of foot bone (12 pieces from each foot) verifies our assumption. According to the principle of superposition between the bone's COM and COS, an evaluation method is put forward to evaluate the bone strength. The result from our evaluation indexes is different from those derived from other evaluation methods such as the BMD (bone mineral density).

## Materials and Methods

### Equipment

The test equipment was Brilliance 64-slice Scanner by Philips, Netherlands, provided by Image Processing Center of Zhujiang Hospital. Scan settings were: frame bone tissue; power: 120kv; pixel size: 0.50 mm; layer distance: 0.50 mm. The scanning was conducted along both feet transect, from top to bottom.

### Software

Software applied included a free trial of SMSolver (The Structural Mechanics Solver for Windows, Version 2.5. http://www.civil.edu.cn/sms/). The three-dimensional model was constructed by Mimics (Version 10) and the statistical analysis was performed by SPSS (Version 12) (provided by the Key Laboratory of Biomechanics and Mechanobiology of Ministry of Education).

### Materials

Altogether, we collected data of 384 pieces of bone - both from the volleyballers (with average height, weight and age of 183.94±3.90 cm, 69.80±5.20 kg and 21.88±0.99 yrs, respectively) and wrestlers (with average height, weight and age of 168.00±5.68 cm, 65.52±5.16 kg and 21.00±2.78 yrs, respectively), i.e. 32 pieces of 12 types of bones: calcaneus, talus, navicular, cuboid, lateral cuneiform, intermediate cuneiform, medial cuneiform, first metatarsal, second metatarsal, third metatarsal, fourth metatarsal and the fifth metatarsal.

The subjects were male volleyball players from our institute and male wrestlers from Provincial Sports School. It was confirmed before the test that every subject had been trained as a professional player for more than five years. Before the test, each subject's medical history was inquired and all the subjects were x-rayed to exclude subjects with diseases such as foot pathological change, deformity or injury to make sure that their physical conditions meet the requirements of the test.

### Definition of the concept

Consider the volume element's (VE) position coordinates (*x,y,z*) with respect to equipment coordinate system. g stands for VE's gray value, *N* the number of VE of the bone, *M* the number of VE of the cross-sectional image. With the help of the following equation, bone's physical quantities such as the COM or COS are defined by the following equation.

The bone's density is defined as
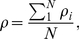
(1)where 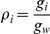, 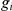 stands for the gray value of the i-th VE, 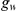 stands for the gray value of water. The equipment has been calibrated, the gray value of the air is set to 0 and that of the water is 1024.

The bone's COS is defined as
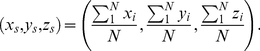
(2)


The bone's COM is defined as
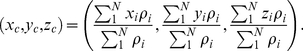
(3)


The distance between the bone's COS and COM is
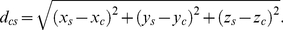
(4)


To the CT data of bone, let's set 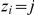. When *j* is set as a constant value, then 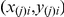 stands for the collection of the *j*-th cross-sectional VE, 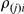 for the density of VE, 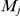 the number of cross-sectional VE. Calculate the cross-sectional image COS by 
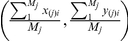
, its COM by 
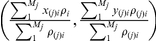
 and the distance between the two points 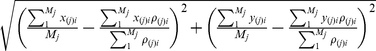.

The bone tissue's radius is
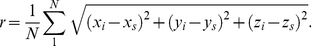
(5)


The same density tissue radius is
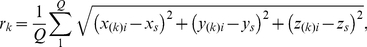
(6)where 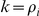. When k is set as a constant value, it refers to the same density tissue of the bone. For example, when 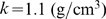, 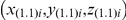, it indicates the VE's coordinates of a density of 1.1. *Q* is the number of VEs when the density is 1.1.

### Ethics Statement

The study received approval from the Ethical Committee of Guangzhou Institute of Physical Education. The subjects provided fully informed consent to participate in this study by signing a written consent form.

## Results and Discussion

Following [15], [16], we separated foot bone to calculate the volume, surface area and BMD. The results for the extracted measurements are shown in Table 1.

**Table 1:** Foot bone volume, surface area and bone density (Mean ±SD).

Item	Wrestler			Volleyballer		
	Volume	Area	Density	Volume	Area	Density
Calcaneus	71.01±8.46	107.39±8.83	1.47±0.04	83.94±6.05	120.70±5.56	1.49±0.05
Talus	38.30±4.33	71.38±5.41	1.63±0.04	43.87±3.33	80.11±5.97	1.65±0.04
Navicular	11.45±1.39	31.21±2.73	1.56±0.04	13.44±1.51	34.78±3.00	1.58±0.05
Cuboid	13.87±1.61	33.14±2.77	1.46±0.04	15.09±2.69	35.24±4.78	1.47±0.05
Lateral cuneiform	5.91±0.69	19.09±1.56	1.51±0.04	6.79±0.61	20.99±1.28	1.53±0.06
Intermediate cuneiform	4.43±0.66	15.69±1.56	1.59±0.04	5.20±0.44	17.56±1.00	1.64±0.06
Medial cuneiform	10.76±1.48	28.60±2.73	1.52±0.03	12.20±1.04	31.02±1.91	1.58±0.05
First metatarsal	16.94±2.23	44.90±3.89	1.62±0.05	20.93±2.25	51.94±3.47	1.65±0.05
Second metatarsal	9.01±1.29	33.72±3.25	1.73±0.07	11.65±0.77	40.56±2.08	1.76±0.08
Third metatarsal	7.72±0.58	30.23±1.60	1.70±0.05	8.99±1.07	34.50±2.61	1.68±0.07
Fourth metatarsal	7.47±0.78	28.80±2.19	1.66±0.04	8.88±0.92	32.97±2.05	1.66±0.05
Fifth metatarsal	8.83±1.09	30.92±2.64	1.72±0.05	9.65±1.07	33.73±2.49	1.71±0.05

Volume is *cm* ^3^, area is *cm* ^2^ and density is *g/cm* ^3^.

BMD is an important index to analyze bone strength. Table 1 shows that no significant difference exists in the foot bone of both groups of athletics. Is that true?

**Figure 1:**
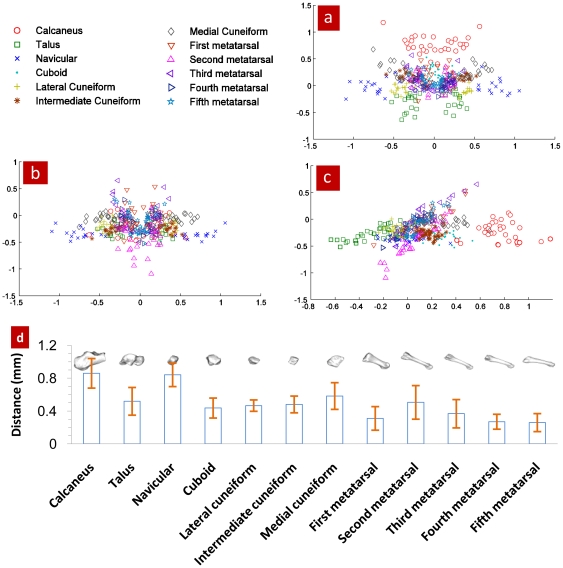
Positional relationship between a bone's COM and COS. Fig. 1a Positional relationship between COM and COS on *x-y* plane; Fig. 1b Positional relationship between COM and COS on *x-z* plane; Fig. 1c Positional relationship between COM and COS on *y-z* plane; Fig. 1d Distance between bone's COM and COS. The bones' COS and COM are derived from the calculation of Eqs. (2) and (3). When choosing coordinate system with origin at COM, the coordinates of COS relative to COM can be derived as 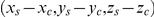. By using 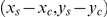, 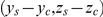 and 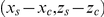, 384 pieces' bone coordinates of COS with respect to COM can be located on *x-y*, *y-z* and *x-z* planes. See Fig. 1a, 1b and 1c (unit is *mm*). Through Eq. (4), the distance of these 384 pieces of bones' COS to the COM can be calculated, resulting in Fig. 1d.

The COM and COS of homogeneous materials superpose exactly one another while those of non-homogeneous materials do not. Bone is a typical non-homogeneous material [17], [18], [19]. Using CT scanning, bone will be separated into a collection of finite VE. The coordinates of each VE and gray value can be provided [16], [20]. This makes it easy to calculate the bone's COM and COS. Setting the bone's COS as the coordinate origin, the positional relationship between the bone's COM and COS can be established. See Fig. 1.

It is known that significant difference exists in the size, density and shape of the navicular and calcaneus. However, Fig. 1 shows that there is no significant difference (p>0.05) in the positional superposition of the COM and COS of both. Shape similarity does exist between the first and second metatarsal, but there is significant difference (p<0.01) in the positional superposition of the COM and COS of both. Therefore, within the range of measurement accuracy, the phenomenon of superposition does exist in the positions of the COM and COS of non-homogeneous bone. It is furthermore unaffected by such different factors as bone size, density or shape.

**Figure 2:**
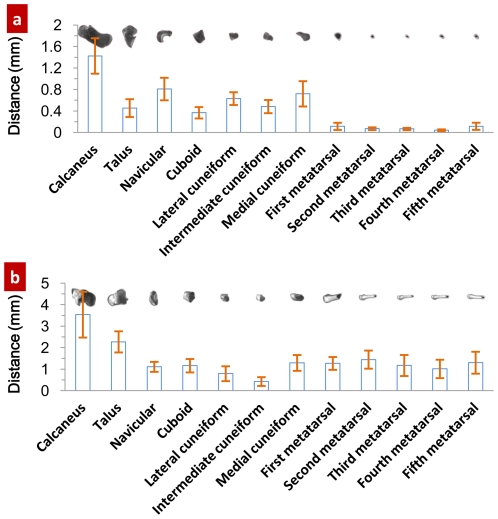
Relationship between the COM and COS of the cross-sectional image. Fig. 2a Positional relationship between the COM and COS of the cross-sectional image through the coordinate origin; Fig. 2b Positional relationship between the COS of the cross section and the COS of the bone. When the position value of the cross-sectional VE at z-axis is approximately equal to the bone's COS, i.e. 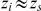, the cross section is the tomography that goes through the bone's COS. Calculate the bone's cross-sectional COM and COS, and then calculate the distance between the two points by using the plane distance formula. See Fig. 2a. Fig. 2b is the distance between the cross-sectional COS and the COS of bone 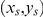 on *x-y* plane calculated by the plane distance formula.

When the cross section passes through the COS of a symmetrical geometry, the COS of the cross section and the COS of the geometry are in the same position. Setting the coordinate origin as the bone's COS, the relationship between the COM and COS of the cross-sectional image through the coordinate origin is set up. See Fig. 2.

Fig. 2a suggests that the COM and COS of the cross-sectional image through the COS of the bone also superpose. Fig. 2b shows difference in the COS position of the cross section and that of the whole bone. Fig. 1 and 2 show that superposition of COM and COS does not only exist in the whole bone, but also in the cross section. Attention should be paid to the fact that it is risky to determine the bone's COS by the cross section's COS since the bone's shape is asymmetric [21].

**Figure 3:**
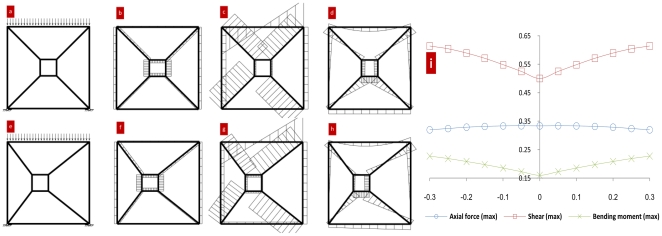
COM and COS of the truss. Fig. 3a and 3e Structure by constraints and loads; Fig. 3b and 3f Axial force distribution in the structure; Fig. 3c and 3g Shear distribution in the structure; Fig. 3d and 3h Bending moment distribution in the structure. Fig. 3i Relationship between internal square position and strength. The rods in the structure are all rigid and the connections between the rods are rigid also. Two squares are drawn with a side length of 1 and 0.2 respectively. Connect the vertices of the two squares and a simple structural mechanics model is forged. Set the two bottom vertices of the bigger square to connect with the hinge bearing on the ground. The top of the bigger square is subjected to distributed load (size is 1). The vertical coordinate of the smaller square COS superposes the bigger square. Change the horizontal coordinate from −0.3 to +0.3. By using the software of **SMSolver**, the calculation results are shown in Fig. 3a–3i.

The bone is then simplified to a truss structure, which is composed of an external square and an internal one. The external square refers to the cortical bone and the internal one to the cancellous bone. When the load and constraint remain the same, the structure strength changes when the position of the internal square changes. See Fig. 3.

Fig. 3 shows that bearing the same constraint and load, the structure where the COS of the internal square superposes with the COS of the external square is superior in the load-carrying capacity of shear and moment to the structure where there is not such a superposition. Therefore, when the force action line of the balance forces passes the COM of an object, the carrying capacity of the structure reaches its maximum.

Though the shape and structure of bone are more complicated than the truss in Fig. 3, the constraints and loads born by the bone *in vivo* are the same as the structure in Fig. 3 – they are both acted upon by out-of-balance forces. It can thus be assumed that when the COS (determined by the bone's shape) is in the same position as the COM (determined by the bone's density distribution), the bone's structure has optimal strength.

It can be concluded that to meet its functional requirements [22], the bone's size, shape [23] and density [22] all produce adaptive changes [3], [5], [6]. In this process, the principle of optimal structure where the COM superposes with COS is always followed. This holds the same idea that function determines the structure as that of the maximal strength with minimal materials [24], or mechanic stability theory [23] or the bone adaptation as an optimization process [6] and Wolf's law [8] (i.e. law of bone transformation) while the superposition of COM and COS is a quantitative description.

**Figure 4:**
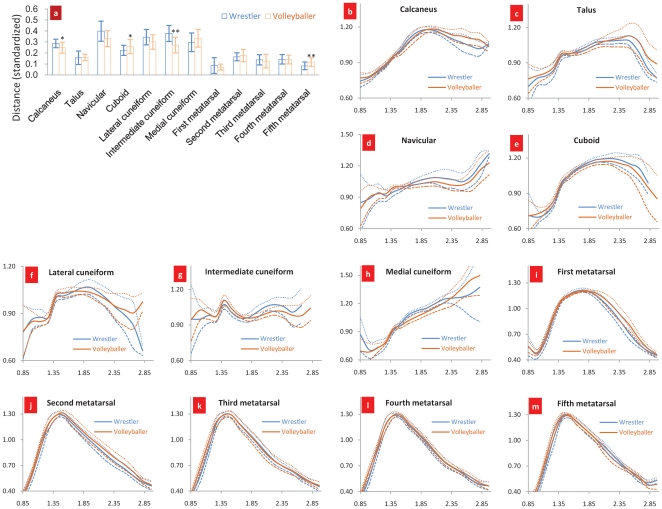
Application of the superposition principle of the bone's COM and COS. Fig. 4a Positional relationship between the COS of the compact bone and the COS of the bone; Fig. 4b–m Relationship between the bone tissue's density and distribution radius, where axis x stands for the tissue's density and axis y for the standardized mean distribution radius of the tissue. The data were collected from 192 pieces of foot bone of the wrestlers and 192 ones of the footballers. *p<0.05, **p<0.01. When 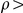
1.65, the bone tissue is defined as compact bone. Eqs (2) and (3) are used to calculate the compact bone's COM and COS while Eq. (4) the distance between the two points and Eq. (5) the distribution radius of bone tissue. Fig. 4a is the result of the distance between the compact bone's COM and COS standardized by the bone tissue's radius. Eq. (6) is applied to calculate same density tissue radius. Then standardize it by the bone tissue's radius. See Fig. 4b–4m.

Why is the superposition of COM and COS a quantitative description? The following relationship can be established based upon the fact that the strength of compact bone is many times greater than that of the spongy bone [25], [26], that the density distribution (relative to the bone's COS) of bone tissue is related to the bone's strength: 1) the relationship between the COS of the compact bone and the COS of the bone where the distance from the compact bone's COS to the bone's COS is standardized by the bone tissue's radius; 2) the relationship between the bone tissue's density and the distribution radius (relative to that of the bone's COS) where the same density tissue radius is standardized by the bone tissue's radius. See Fig. 4.

In Fig. 4a, the distance of the volleyballers' calcaneus compact bone's COS to the bone's COS is shorter than that of the wrestlers and it has a significant difference (p<0.05), which is in contrast with estimates in Table 1 where the no significant difference is observed. A similar trend is observed for the distance of fifth metatarsal compact bone's COS to the bones' COS. From Table 1, we can see that in the similar morphological first to fifth metatarsal, the lowest density goes to the first metatarsal, which does not sound very reasonable, suggesting the limitation of bone density assessment index, i.e. factors such as volume and joint segmental area might have affected bone density. In Fig. 4a, the distance of both athletic groups' first metatarsal compact bone's COS to the bone's COS is the shortest.

When a volleyballer takes off to spike, the braking movement has a great impact on the calcaneus. In Fig. 4b, the distribution radius of the volleyballers' calcaneous begins to become larger than that of the wrestlers from the density of compact bone on; especially when comparing this with the results from the marrow and spongy bone tissues (when density 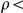
1.14, it is the marrow; when 1.14
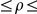
1.65, the spongy bone and when 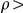
1.65, the compact bone), this difference is outstanding. The wrestlers' fierce body combats carry great strength to their fifth metatarsal from the front, rear, left and right. The distribution radius of the wrestlers' fifth metatarsal begins to become bigger from the density of compact bone on than that of the volleyballers.

Fig. 4 shows that according to the superposition principle of the bone's COM and COS, the establishment of relationship between the compact bone's COS and the bone's COS and the relationship between the tissue's density and distribution radius has offered a new approach to study the bone's strength.

**Figure 5:**
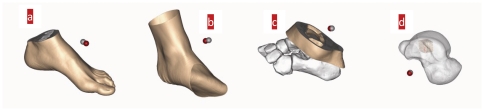
COM and COS of non-bone tissues. Fig. 5a Positional relationship between the whole foot's COM and COS; Fig. 5b Positional relationship between the COM and COS of ankle skin; Fig. 5c Positional relationship between the COM and COS of non-bone tissues (a group of cross sections selected around the ankle joint); Fig. 5d Positional relationship between the COM and COS when a ROI (region of interest) of 1 *cm* ^3^ is established around the COS of the talus. The grey ball stands for the COM position and the red ball for the COS position. The radius of the ball is 0.5 *mm*. According to the definition of non-bone tissue density, Eqs. (2) and (3) are used to calculate COM and COS of non-bone tissue. The three-dimensional model is constructed by the software of **Mimics**.

What insight will this phenomenon of superposition between the COM and COS bring to biomechanical research? Using the CT data of bone, we analyze the COM and COS of the other foot non-bone tissues. See Fig. 5.

Fig. 5 shows that the COM and COS of the whole foot, of its ankle skin, of the non-bone tissues around the ankle joint and of the ROI established around the whole bone's COS superpose highly. Further subdivisions tell us that if the COM and COS of the cell also follow this principle of high superposition, then a new method of dynamics can be set up to study activities such as cell growth and division.

The COS of a continuous closed geometry superposes with that of its surface (shape). The COS of the cell can thus be obtained through the numerical model of the cell surface. According to the dynamic principle of COM (i.e. the internal force cannot change the motion of the system's COM), if the forces acting on the cell are known, the cell's kinematic characteristics can be obtained. On the other hand, we can use the kinematic characteristics of the cell to analyze the characteristics of external mechanical signals. When the cell shape is asymmetrical, its geometric transformation invariance and the uniqueness of the principal moments of inertia axes [27], [28] can be applied to study issues such as the rotational dynamics of the cell.

### Conclusion

The physiological activities of the bone are a process of optimization. In this adaptive changing process, what remains unchanged is the optimal structure principle of superposition of COM and COS. The mechanical significance of following the optimal structure principle is to use the optimal structure to bear the external load.

We propose the concept of distance between the tissue's COS and its bone's COS and discover the relationship between the distance (of the compact bone's COS and its bone's COS) and the loading type. This relationship is represented by the phenomenon that the impact strength has made the compact bone's COS move towards the bone's COS. This movement symbolizes a functional adaptation of bone in its structure. The physiological activity of the middle aged and seniors is mostly a reconstruction [29]. When their bone masses are gradually decreasing, it is essential to look into the possibility of whether physical exercises can diminish the bone loss and change the movement's direction. This is meaningful and worthwhile research.

With the advances of three-dimensional imaging technology [30], [31], if this phenomenon of superposition of COM and COS also happens in cell, this will play a significant role in the study of cytokinetics.
